# Supplementary material for: TGFβ reprograms TNF stimulation of macrophages towards a non-canonical pathway driving inflammatory osteoclastogenesis
Source: Nat Commun. 2022 Jul 7;13:3920. doi: 10.1038/s41467-022-31475-1 (PMC9263175; doi:10.1038/s41467-022-31475-1)
Supplement: Supplementary file 1 — Supplementary information [file 41467_2022_31475_MOESM1_ESM.pdf]

Supplementary Fig. 1

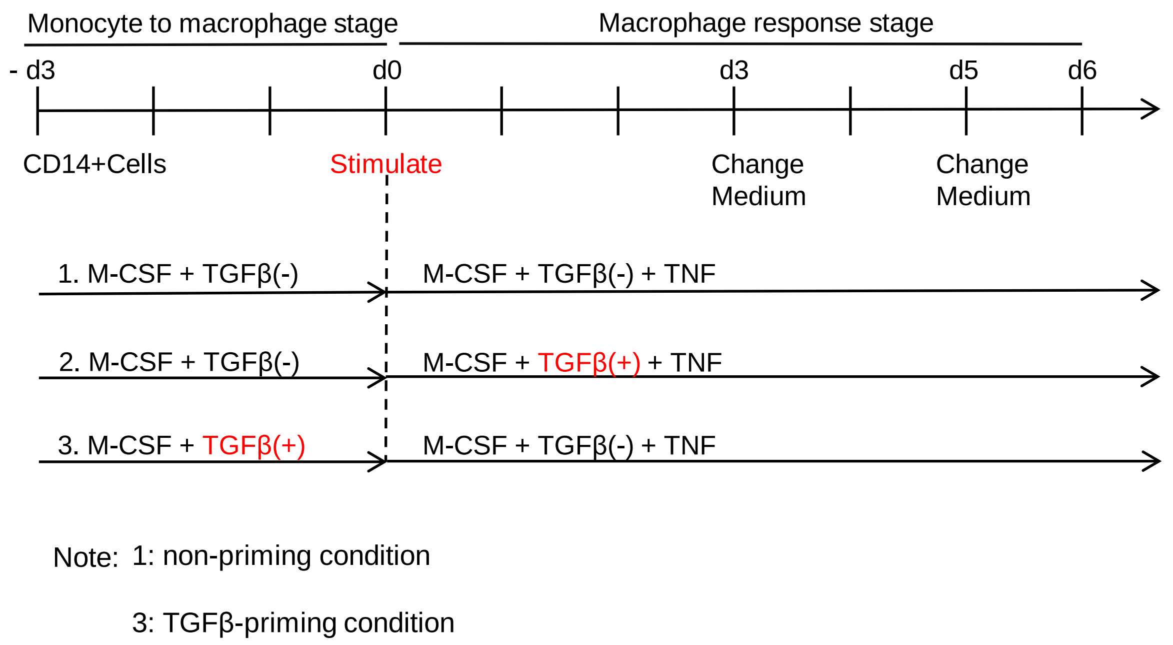

Supplementary Fig. 1 Schematic of experimental design of the human cell culture system used in Fig.1a.

Supplementary Fig. 2

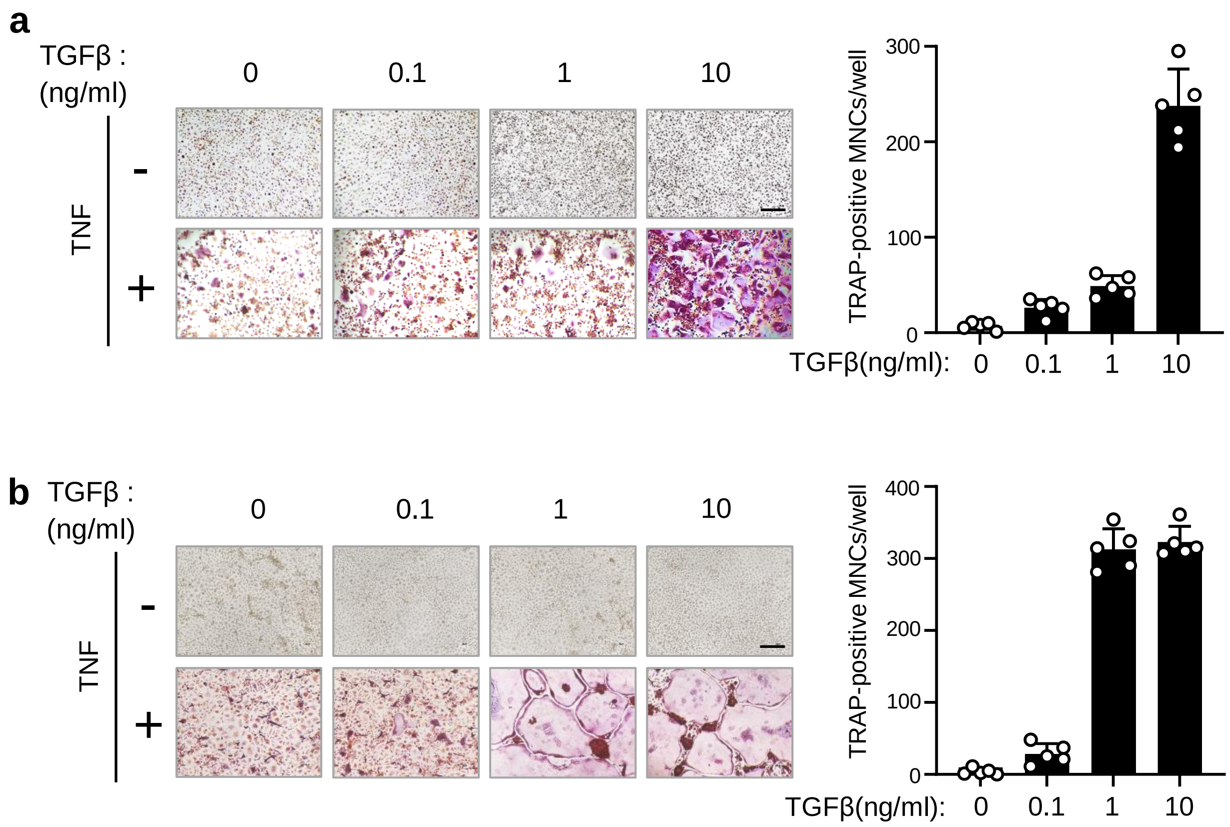

Supplementary Fig. 2 Dose dependent effects of TGFβ priming on TNF-induced osteoclastogenesis using human CD14(+) monocytes (**a**) or mouse bone marrow derived macrophages (**b**). Osteoclast differentiation was determined by TRAP staining (left panel) and the relative area of TRAP-positive multinuclear osteoclasts (MNCs, ≥3 nuclei/cell) per well (right panel) (n = 5/group). **a, b,** Data are mean ± SD. Scale bars: 200 µm. Source data are provided as a Source Data file.

Supplementary Fig. 3

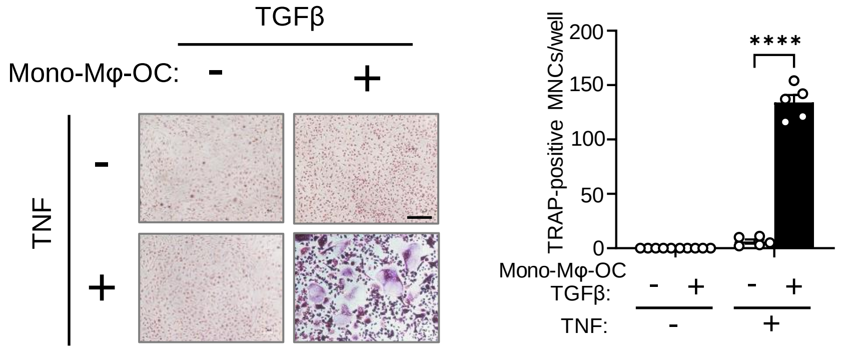

Supplementary Fig. 3 Human osteoclast differentiation determined by TRAP staining (left panel) and the relative area of TRAP-positive multinuclear osteoclasts (MNCs, ≥3 nuclei/cell) per well (right panel) in the cell cultures using human CD14(+) monocytes treated with or without TGFβ for three days. The cells were then treated by TNF for six days without removal of TGFβ or in the absence of TGFβ. Mono-Mφ-OC: monocyte to macrophage to osteoclast stage. TRAP-positive cells appear red in the photographs (n = 5/group). ****$p < 0.0001$ by two-way ANOVA with Bonferroni's multiple comparisons test. Data are mean ± SD. Scale bars: 200 µm. Source data are provided as a Source Data file.

Supplementary Fig. 4

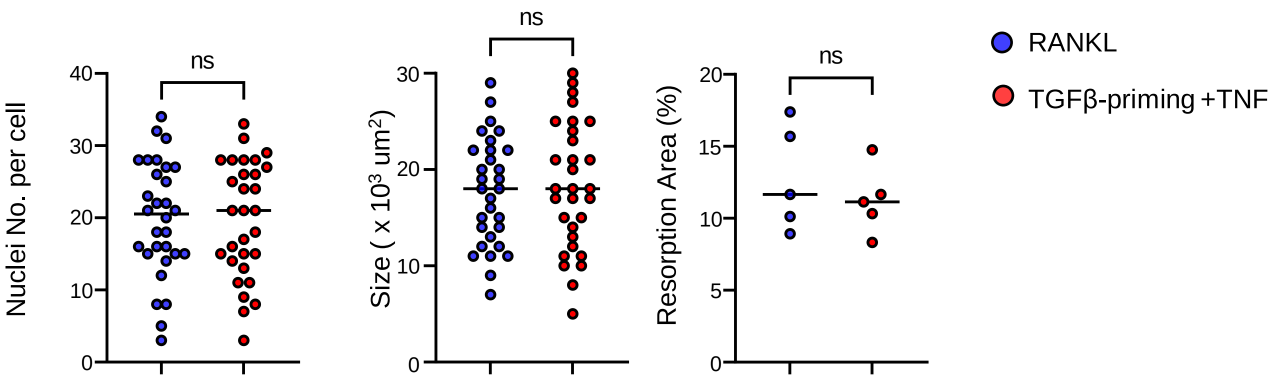

Supplementary Fig. 4 Comparison of the number of nuclei (left panel), the size (middle panel) and the resorption area (%) (right panel) of human RANKL-induced osteoclasts and TGFβ priming/TNF-induced osteoclasts in cultures. The number of nuclei and the size (n=30 from 5 donors), as well as the resorption area (n= 5 donors) were determined by Image J.

ns, not statistically significant by two-sided Student's t test. Error bars: Data are mean ± SD. Source data are provided as a Source Data file.

Supplementary Fig. 5

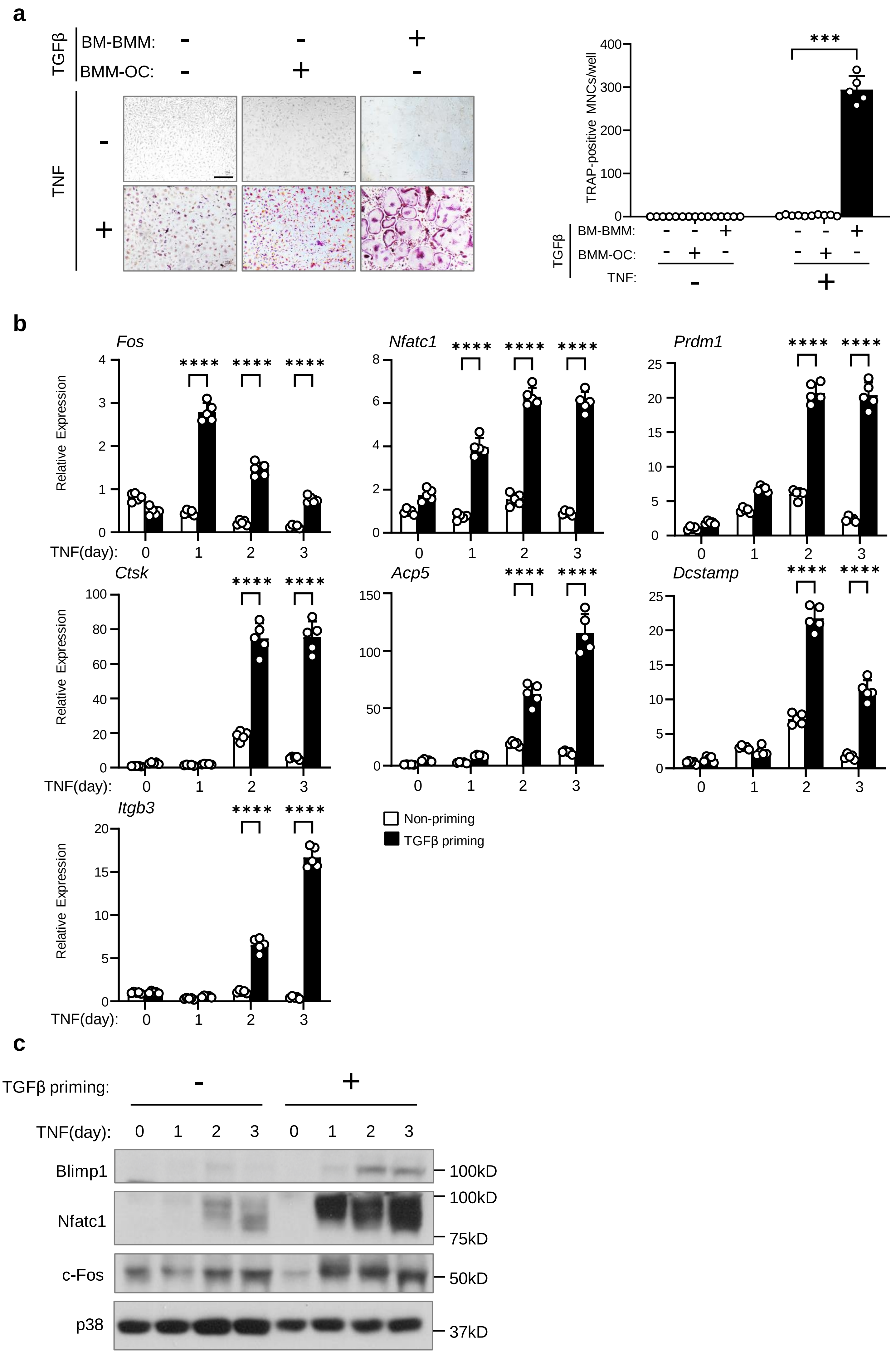

Supplementary Fig. 5 TGFβ priming switches the action of TNF to effectively drive mouse osteoclastogenesis.
(**a**) Mouse osteoclast differentiation determined by TRAP staining (left) and the relative area of TRAP-positive MNCs (≥3 nuclei/cell) per well (right) in the cell cultures, in which the bone marrow was primed with or without TGFβ for four days, followed by TNF stimulation for three days in the presence or absence of TGFβ. TRAP-positive cells appear red in the photographs. (n = 5/group)
(**b-c**) qPCR analysis of mRNA expression of *Fos*, *Nfatc1*, *Prdm1*, *Ctsk*, *Acp5*, *Dcstamp*, and *Itgb3* (**b**), *and* immunoblot analysis of the expression of Blimp1, Nfatc1, and c-Fos (**c**) in the cell cultures, in which the bone marrow was primed with or without TGFβ for four days, followed by TNF stimulation for the indicated times. p38 was used as a loading control. (n = 5/group)
a, **b** ***p < 0.001; ****p < 0.0001 by two-way ANOVA with Bonferroni's multiple comparisons test. Error bars: **a**, **b** Data are mean ± SD. Scale bars: **a** 200 µm. Source data are provided as a Source Data file.

Supplementary Fig. 6

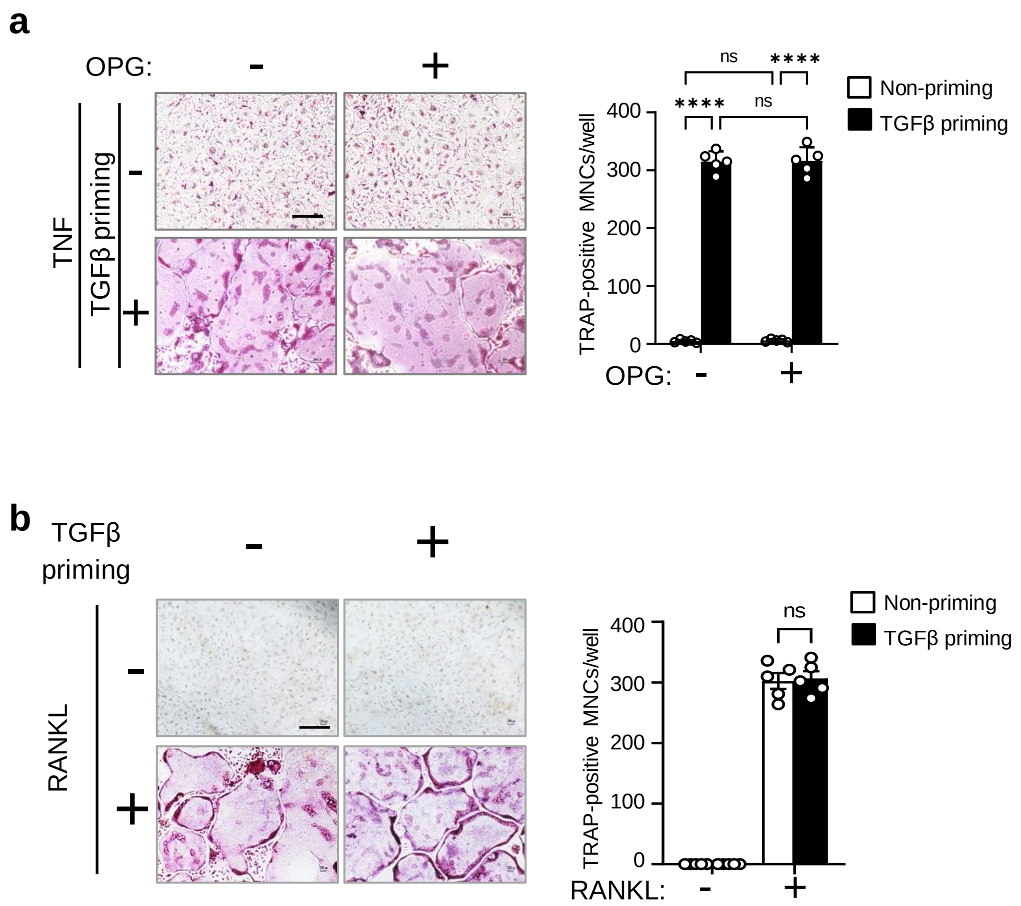

Supplementary Fig. 6 TGFβ priming and TNF-induced osteoclastogenesis is independent of RANKL. **(a**) Mouse TNF-induced osteoclastogenesis in the cell cultures, in which the bone marrow was primed with or without TGFβ for four days, followed by TNF stimulation for three days in the presence or absence of recombinant OPG (100 ng/ml). Left panel: TRAP staining; Right panel: quantification of the relative area of TRAP-positive MNCs per well. (n = 5/group)
(**b**) Mouse RANKL-induced osteoclastogenesis using bone marrow derived macrophages treated with or without TGFβ priming for three days, followed by RANKL stimulation for four days. Left panel: TRAP staining; Right panel: quantification of the relative area of TRAP-positive MNCs per well (n = 5/group).
****$p < 0.0001$; ns, not statistically significant by two-way ANOVA with Bonferroni's multiple comparisons test. Error bars: Data are mean ± SD. Scale bars: 200 µm. Source data are provided as a Source Data file.

Supplementary Fig. 7

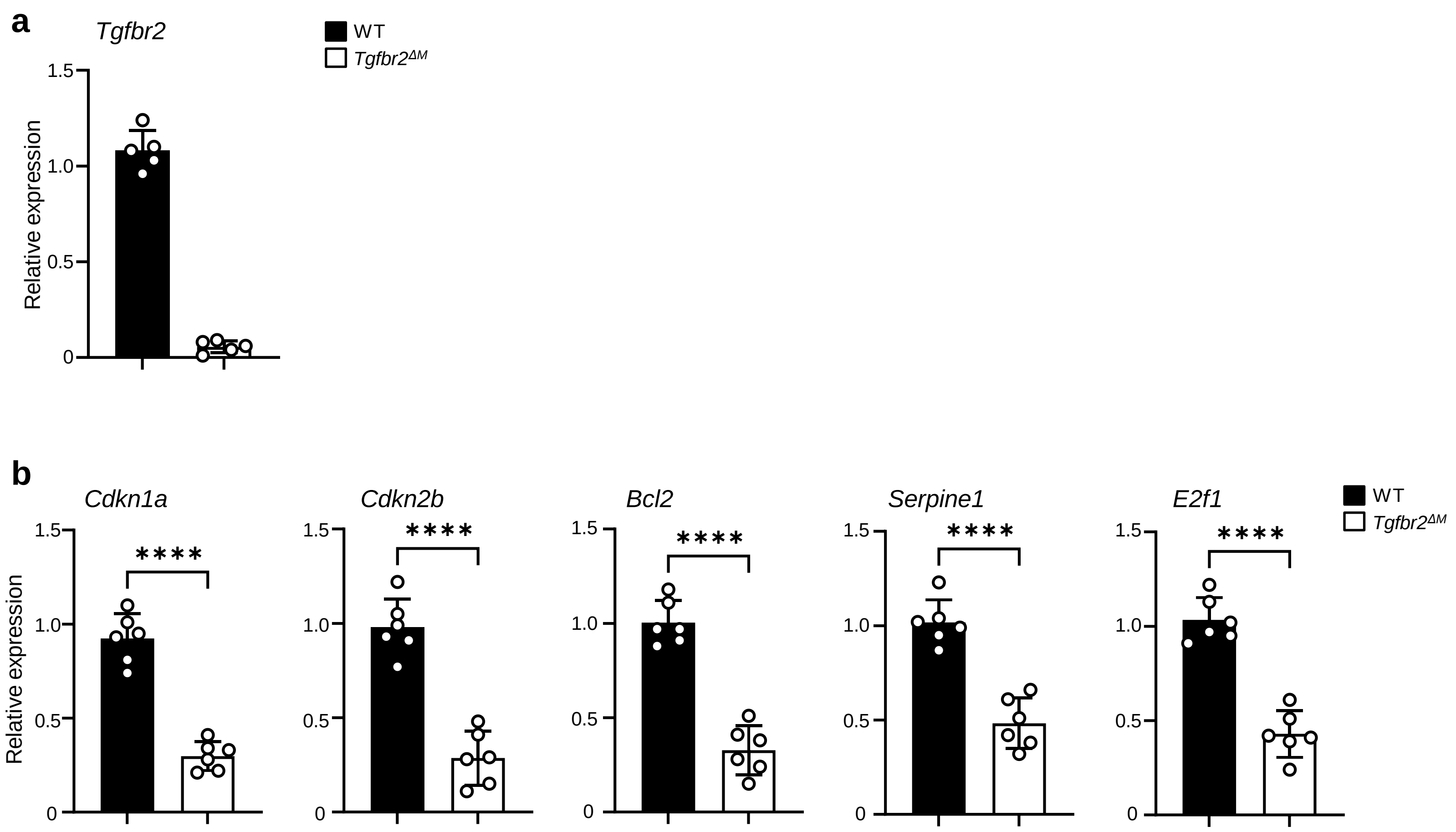

Supplementary Fig. 7 The expression of *Tgfbr2* and TGFβ signaling target genes in *Tgfbr2$^{\Delta M}$* BMMs.
(**a**) qPCR analysis of mRNA expression of *Tgfbr2* using WT and *Tgfbr2$^{\Delta M}$* BMMs. (n = 5/group)
(**b**) qPCR analysis of mRNA expression of TGFβ signaling target genes, including *Cdkn1a, Cdkn2b, Bcl2, Serpine1 and E2f,* using WT and *Tgfbr2$^{\Delta M}$* BMMs. (n = 6/group). **a, b,** Data are mean ± SD. **b** ****p < 0.0001 by two-sided Student's t test. Source data are provided as a Source Data file.

Supplementary Fig. 8

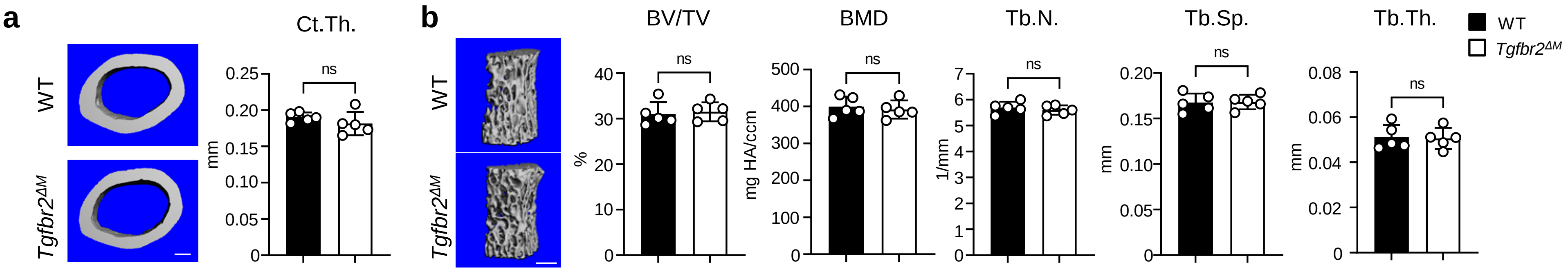

Supplementary Fig. 8 *Tgfbr2*$^{\Delta M}$ mice do not exhibit significant defects in bone phenotype of femoral trabecular and cortical bones and vertebral trabecular bones.

(**a**) µCT images (left panel) and cortical thickness (right panel) of the midshaft of femurs isolated from 12-week-old male WT and *Tgfbr2*$^{\Delta M}$ littermate mice (n = 5/group).

(**b**) µCT images (left panel) and bone morphometric analysis (right panel) of trabecular bone of the L5 vertebrae isolated from 12-week-old male WT and *Tgfbr2*$^{\Delta M}$ littermate mice (n = 5/group).

a, **b** ns, not statistically significant by two-sided Student’s t test. Error bars: **a**-**g** Data are mean ± SD. Scale bars: **a** 200 µm; **b** 500 µm. Source data are provided as a Source Data file.

Supplementary Fig. 9

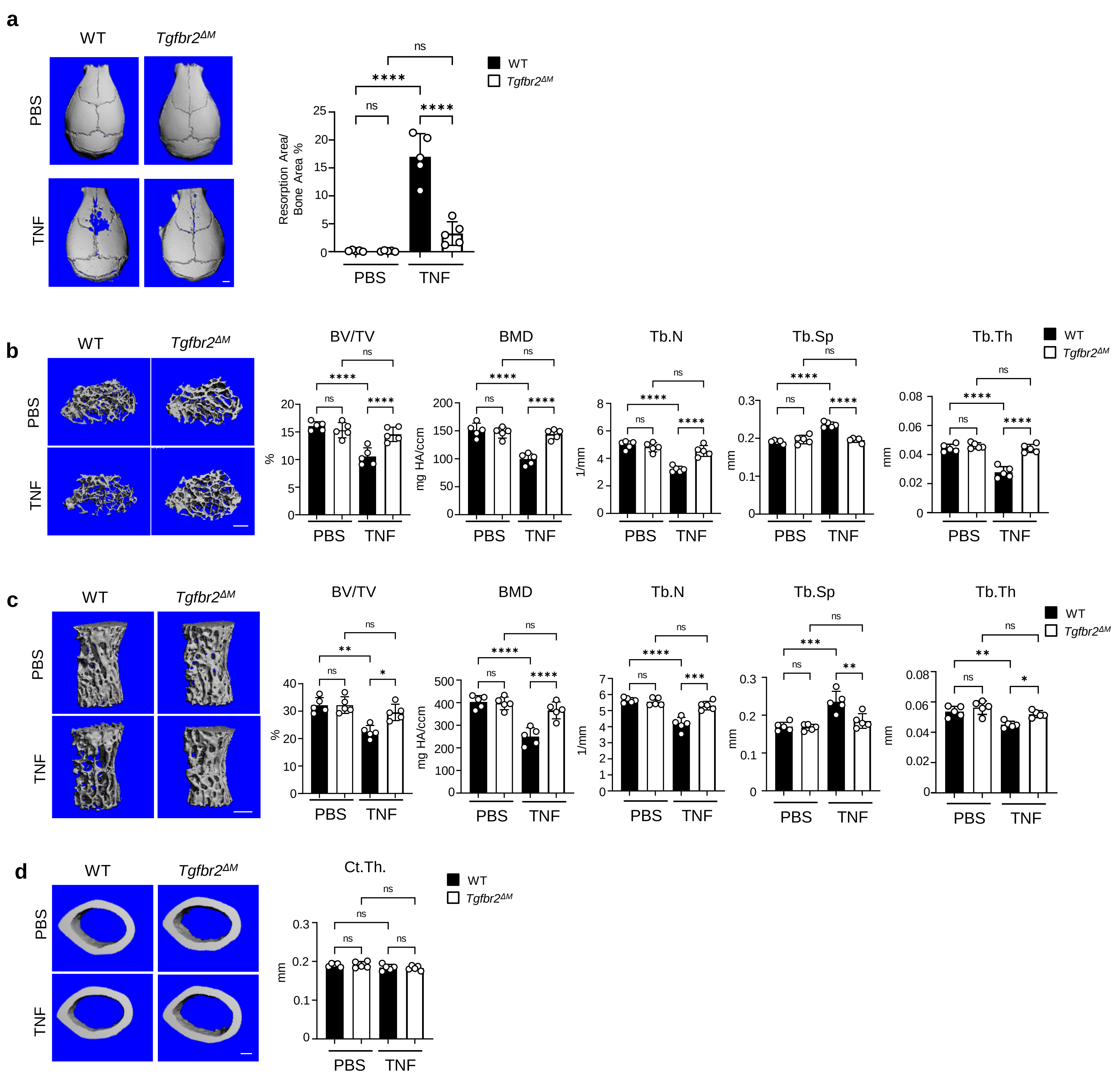

Supplementary Fig. 9 TGFβ signaling plays a critical role in enhancing TNF-mediated inflammatory bone loss.
(**a**) µCT images (left panel) and the quantification of the resorption area (right panel) of the calvarial bones obtained from 14-week-old male WT and *Tgfbr2*$^{\Delta M}$ mice after PBS or TNF injection to the calvarial periosteum daily for fourteen days (n= 5/group).
(**b**) µCT images (left panel) and bone morphometric analysis (right panel) of trabecular bone of the distal femurs isolated from 14-week-old male WT and *Tgfbr2*$^{\Delta M}$ after PBS or TNF injection to the calvarial periosteum daily for fourteen days (n= 5/group).
(**c**) µCT images (left panel) and bone morphometric analysis (right panel) of trabecular bone of the L5 vertebrae isolated from 14-week-old male WT and *Tgfbr2*$^{\Delta M}$ after PBS or TNF injection to the calvarial periosteum daily for fourteen days (n= 5/group).
(**d**) µCT images (left panel) and cortical thickness (right panel) of the midshaft of femurs isolated from 14-week-old male WT and *Tgfbr2*$^{\Delta M}$ after PBS or TNF injection to the calvarial periosteum daily for fourteen days (n= 5/group).
a, **b**, **c**, **d** *p < 0.05; **p < 0.01; ***p < 0.001; ****p < 0.0001; ns, not statistically significant by two-way ANOVA with Bonferroni's multiple comparisons test. Error bars: **a-d** Data are mean ± SD. Scale bars: **a** 1.0 mm; **b** 100 µm; **c** 500 µm; **d** 200 µm. Source data are provided as a Source Data file.

Supplementary Fig. 10

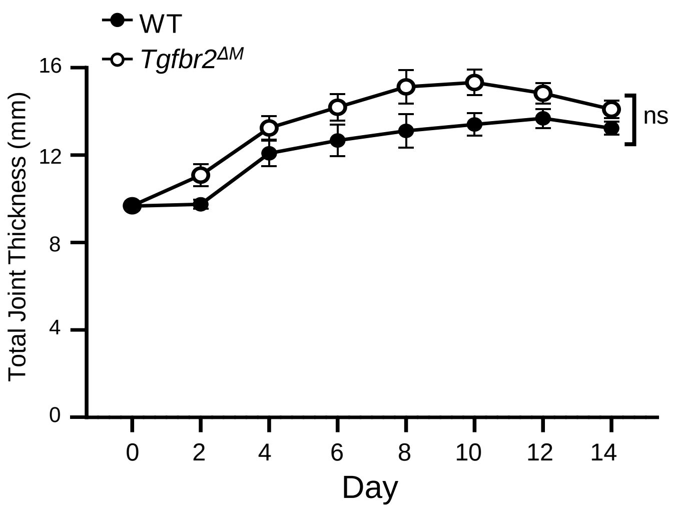

Supplementary Fig. 10 Time course of joint swelling of inflammatory arthritis developed in *Tgfbr2*$^{\Delta M}$ mice and littermate controls. For each mouse, joint swelling was calculated as the sum of measurements of joint thickness of two wrists and two ankles (n = 6/group). Joint swelling is represented as the mean ± SEM for each group.
n.s., not statistically significant by 2-way ANOVA with Bonferroni's multiple comparisons test. Source data are provided as a Source Data file.

Supplementary Fig. 11

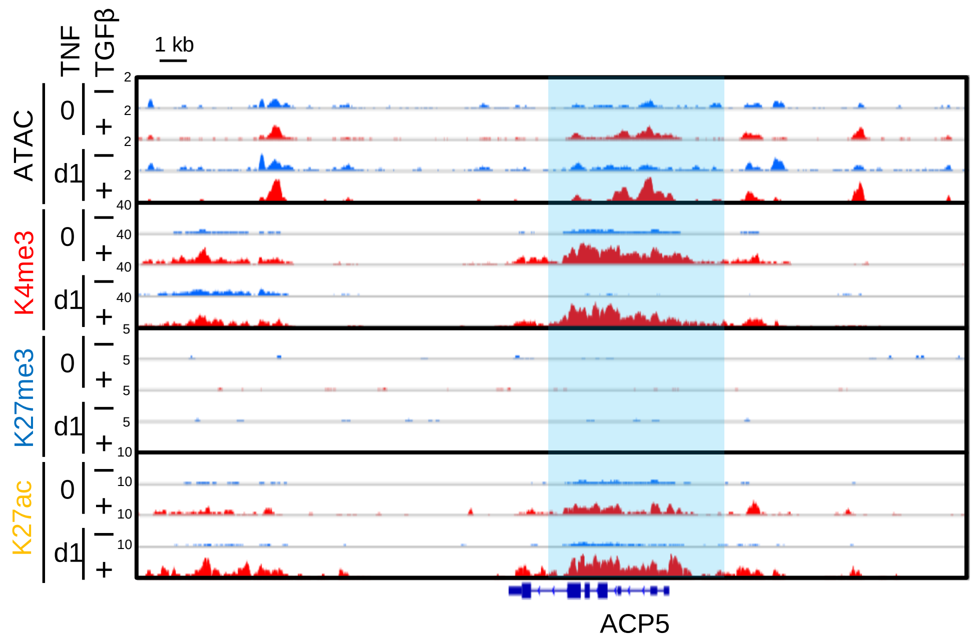

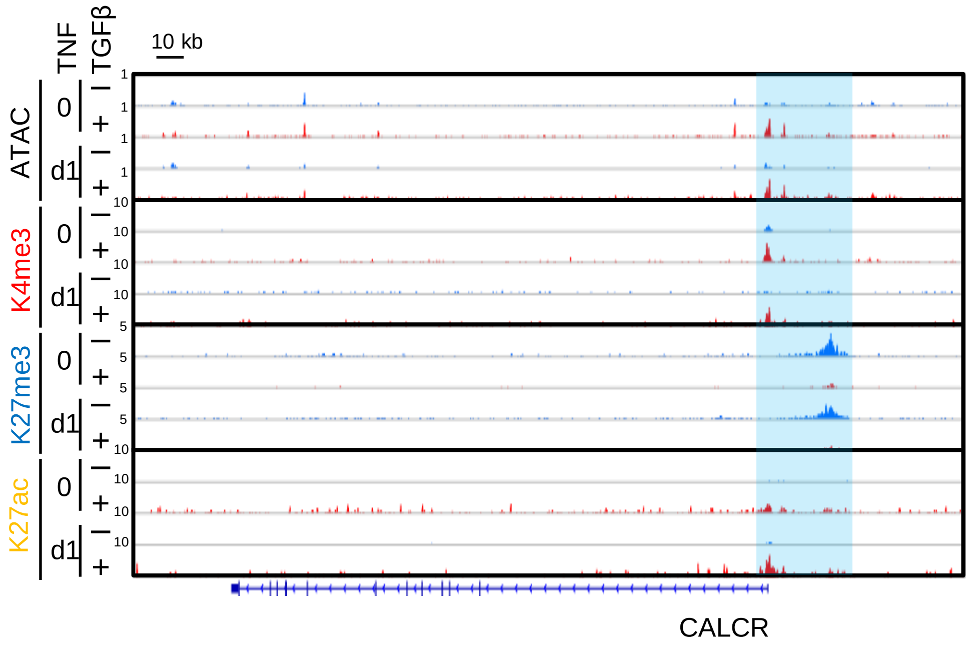

Supplementary Fig. 11 Representative Integrative Genome Browser tracks displaying normalized tag density profiles for ATAC-seq, H3K4me3, H3K27me3 and H3K27ac Cut&Run-seq signals at the indicated osteoclast gene loci. Data are representative of 2 biological replicates.

Supplementary Fig. 12

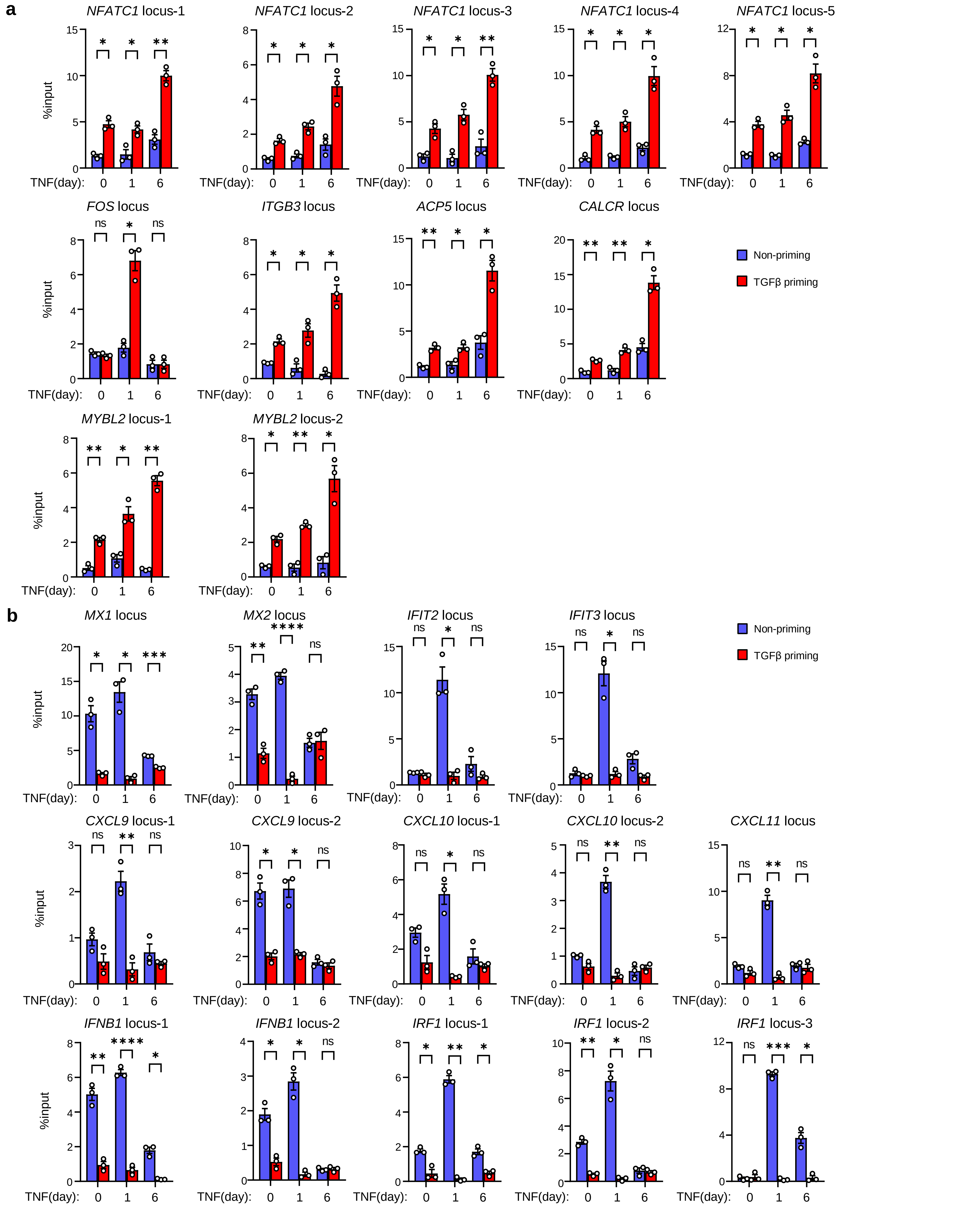

Supplementary Fig. 12 Chromatin accessibility of OC gene loci (a) and ISG gene loci (b).
Chromatin accessibility of *NFATc1*, *FOS*, *ITGB3*, *ACP5*, *CALCR*, *MYBL2*, *MX1*, *MX2*, *IFIT2*, *IFIT3*, *CXCL9*, *CXCL10*, *CXCL11*, *IFNB1* and *IRF1* loci during osteoclastogenesis using human CD14(+) monocytes treated with or without TGFβ priming for 3 days, followed by TNF stimulation for the indicated days. Chromatin accessibility was measured by FAIRE-qPCR. Chromatin accessibility is displayed relative to total input. n=3/group. Data are mean ± SEM. *$p < 0.05$; **$p < 0.01$; ***$p < 0.001$; ****$p < 0.0001$; ns, not statistically significant by two-way ANOVA with Bonferroni's multiple comparisons test. Source data are provided as a Source Data file.

Supplementary Fig. 13

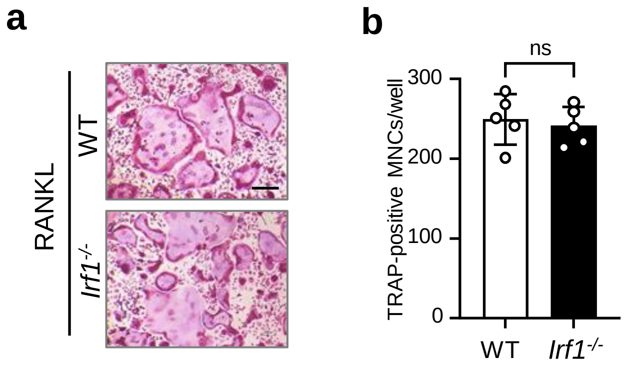

Supplementary Fig. 13 *Irf1* deficiency does not affect RANKL-induced osteoclastogenesis**.** Osteoclast differentiation induced by RANKL for three days was determined by TRAP staining (left panel) and the relative area of TRAP-positive multinuclear osteoclasts (MNCs, ≥3 nuclei/cell) per well (right panel) (n = 5/group). Scale bars: 200 µm. Data are mean ± SD. ns: not statistically significant by two-sided Student’s t test. Source data are provided as a Source Data file.

Supplementary table 1. Primer sets for FAIRE-qPCR

Gene locus	Forward/Reverse	Sequence
MX1	Forward	AGCGAGCAGAAATGAAACCG
	Reverse	ATCCTGCACTGGGCTTACTTC
MX2	Forward	TTGTGTCTGATGCACAGCAG
	Reverse	ACAAGCCACATTCACCAAGC
IFIT2	Forward	GGTGGCAGAAGAGGAAGATTTC
	Reverse	TGCAGTGCAATTCTCAGCTG
IFIT3	Forward	ACTTGAGGCAGACAGGAAGAC
	Reverse	TCTCTGCTGTTCCGAAAAGC
CXCL9 locus-1	Forward	TTCAAACTGCTTGGCTCACC
	Reverse	TAGGCTTCCACGTACTGCAG
CXCL9 locus-2	Forward	TCTGAGATAGCAAGCGGAATGG
	Reverse	TGGCAGAACAGTTTCACCAC
CXCL10 locus-1	Forward	ATTCATGGTGCTGAGACTGGAG
	Reverse	TAGCCCCACGTTTTCTGAGAC
CXCL10 locus-2	Forward	AACTGAGTGCTGGCAAAGTG
	Reverse	AGAAGCTATTCTGAGCCTCTGC
CXCL11	Forward	CAGCGTCCTCTTTTGAACATGG
	Reverse	ACCACCAGCTATAAACCACAGC
IFNB1 locus-1	Forward	GCCAAGGGAAAAACGATGACTC
	Reverse	TTCCCATTTGCTCCTTTGCC
IFNB1 locus-2	Forward	ACTGCTGCAGCTGCTTAATC
	Reverse	AGAAGCTCCTGTGGCAATTG
IRF1 locus-1	Forward	AGGGTTTCAGTCCTAGCACTAC
	Reverse	GCTTTCTGCCTTCTTCACTTCC
IRF1 locus-2	Forward	AGGAGCCTAACTGGAGAGAAAG
	Reverse	TGGGGTTCACCAAAAAGCAG
IRF1 locus-3	Forward	ATGCATGCCTTTGAGACCAC
	Reverse	TCTTTCCAAGAGCCATGGTCTC
NFATC1 locus-1	Forward	ATAAGGGTGTCGTGCAATCG
	Reverse	TCTTGTTGGCTTTGGATGGG
NFATC1 locus-2	Forward	TTCGGGAGAGGAGAAACTTTGG
	Reverse	TTACCTTCCTCCGCTGACTTC
NFATC1 locus-3	Forward	GTCGGCAAAAGCAGCAAAAC
	Reverse	CCGTGTGGCCTGATTTAGTTTC
NFATC1 locus-4	Forward	TCTTCACGGGGTGCTTTGAG
	Reverse	ATTCTTTGCACCTGCTGAGC
NFATC1 locus-5	Forward	TGATCAGGGAAAACGCATGC
	Reverse	TCGATGAGGCAAAGAAACACC
FOS	Forward	AAGGGTAAAAAGGCGCTCTC
	Reverse	ATTCGCACCTGGTTCAATGC
ITGB3	Forward	GCGAGAGAGGAGCAATAGTTTC
	Reverse	TAGATCCCCTCGCGCTTCTC
MYBL2 locus-1	Forward	TGGGAAGTCATGGACCGTTC
	Reverse	AAGAAAGTCGCAGGGATGAGAG
MYBL2 locus-2	Forward	AGTAGTGGCTTGGACTAGCG
	Reverse	ATAGCGAAGACCGAGGAAGG
ACP5	Forward	TGGGAGCTGTGTTTGACAAC
	Reverse	AGCCCGTTGGTGTTTATGTG
CALCR	Forward	CCCTTTGGAGATTGGGACTTTG
	Reverse	AGGGTGGAAATTTGCAGCAG
